# Comparative Assessment of Phytochemical Profiles of Comfrey (*Symphytum officinale* L.) Root Extracts Obtained by Different Extraction Techniques

**DOI:** 10.3390/molecules25040837

**Published:** 2020-02-14

**Authors:** Nataša Nastić, Isabel Borrás-Linares, Jesús Lozano-Sánchez, Jaroslava Švarc-Gajić, Antonio Segura-Carretero

**Affiliations:** 1 Faculty of Technology, University of Novi Sad, Bulevar Cara Lazara 1, 21000 Novi Sad, Serbia; natasa.nastic@uns.ac.rs (N.N.);; 2 Functional Food Research and Development Centre (CIDAF), Health Science Technological Park, Avda. del Conocimiento s/n, Bioregion building, 18016 Granada, Spain; iborras@ugr.es (I.B.-L.); ansegura@ugr.es (A.S.-C.); 3 Department of Food Science and Nutrition, University of Granada, Campus Universitario s/n, 18071 Granada, Spain; 4 Department of Analytical Chemistry, Faculty of Sciences, University of Granada, Avda. Fuentenueva s/n, 18071 Granada, Spain

**Keywords:** comfrey (*Symphytum officinale* L.) root, maceration, PLE, SFE, HPLC-ESI-QTOF-MS/MS, phytochemical compounds

## Abstract

In this work a comparative study on phytochemical profiles of comfrey root extracts obtained by different extraction approaches has been carried out. Chemical profiles of extracts obtained by supercritical fluid (SFE), pressurized liquid (PLE), and conventional solid/liquid extraction were compared and discussed. Phytochemical composition was assessed by high-performance liquid chromatography coupled with electrospray time-of-flight mass spectrometry (HPLC-ESI-QTOF-MS/MS) identifying 39 compounds reported for the first time in comfrey root, mainly phenolic acids and fatty acids. The influence of different extraction parameters on phytochemical profiles of *S. officinale* root was investigated for all applied techniques. PLE and maceration, using alcohol-based solvents (aqueous methanol or ethanol), were shown to be more efficient in the recovery of more polar compounds. Greater numbers of phenolics were best extracted by PLE using 85% EtOH at 63 °C. The use of SFE and 100% acetone for 30 min enabled good recoveries of nonpolar compounds. SFE using 15% EtOH as a cosolvent at 150 bar produced the best recoveries of a significant number of fatty acids. The main compositional differences between extracts obtained by different extraction techniques were assigned to the solvent type. Hence, these results provided comprehensive approaches for treating comfrey root enriched in different phytochemicals, thereby enhancing its bioaccessibility.

## 1. Introduction

Comfrey (*Symphytum officinale* L.) is a medicinal plant widely spread across Europe, but it can also be found in some parts of Asia and South America. In traditional medicine, comfrey roots are used topically, mostly for the treatment of wounds, joint disorders, and musculoskeletal injuries of all kinds due to pyrrolizidine alkaloids that have been linked to hepatotoxicity and carcinogenicity [1,2]. The content of pyrrolizidine alkaloids is the highest in comfrey root [3,4]. Compounds that were identified in comfrey root as active in the treatment of sprains, arthritis, fractures, and hematoma include allantoin, rosmarinic acid, and other hydroxycinnamic acid derivatives, as well as muco-polysaccharides, A, B and C vitamins, triterpenoid saponins, tannins, calcium, potassium, and selenium [5,6]. Allantoin activates metabolic processes in subcutaneous tissue and stimulates cell growth resulting in epithelization. It also strongly promotes the cell growth in bones and connective tissue [7]. 

In the literature, few papers dealt with the extraction of polyphenols from comfrey root relying mostly on conventional solid/liquid extraction [8,9,10,11,12].

Conventional solid/liquid extraction using different solvents is the most widespread procedure for isolation of bioactive compounds from plant material [13,14]. The major disadvantages of conventional solid/liquid extraction are the use of large quantities of organic solvents, long extraction times, and relatively poor selectivity [15]. For these reasons, a new generation of extraction techniques are emerging to overcome these disadvantages. 

Pressurized liquid extraction (PLE) operates at high temperatures (up to 200 ^º^C) and pressures (>1000 psi), which greatly facilitates penetration of the solvent into the solid matrix. Elevated temperatures increase the solubility of the target compound, while decreasing solvent viscosity and surface tension, drastically improving extraction efficiency [13,16,17]. Moreover, during PLE run, target compounds are protected from oxygen and light, which is very important for the extraction of bioactive compounds such as polyphenols.

Nowadays, supercritical fluid extraction (SFE) has become one of the most popular green extraction techniques. Carbon dioxide is the most used supercritical fluid, sometimes modified by cosolvents. The popularity of supercritical CO_2_ is due to its low critical parameters (T_c_ = 31.1 ^º^C and P_c_ = 7.38 MPa), non-toxicity, low price, and availability in high purity. The most important process parameters of SFE (temperature, pressure, and cosolvent) can be optimized for high efficiency and selectivity [18,19]. The process is also characterized with no organic residues and the absence of thermal degradation, resulting in a very high quality of extracts.

Very little information is available on the phytochemical composition of comfrey root. Previous studies have been mostly focused on the recovery of polyphenolic compounds. Savić et al. [10] revealed the presence of phenolic acids, flavonoids, and allantoin. In another report [12], extracts of comfrey root were rich in rosmarinic, caffeic, and salvianolic acids. The profile of comfrey root phenolic compounds has also been reported by Alkan et al. [20], Neagu et al. [21], Roman et al. [9], Sowa et al. [22] and Tahirovic et al. [11]. In order to assess the *S. officinale* root as a potential source of different phytochemicals, it is important to determine a detailed chemical profile. This research focused on the recovery of phytochemical compounds using three different extraction techniques (maceration, PLE, and SFE), and the comparison of chemical profiles in different extracts. Major phytochemical compounds in comfrey root extracts were identified by high-performance liquid chromatography coupled with electrospray time-of-flight mass spectrometry (HPLC-ESI-QTOF-MS). The influence of different extraction parameters on the recovery of phytochemicals from comfrey root was investigated for all three extraction techniques.

## 2. Results and Discussion

### 2.1. Chemical Profiles of Comfrey Root Extracts

**Figure 1:**
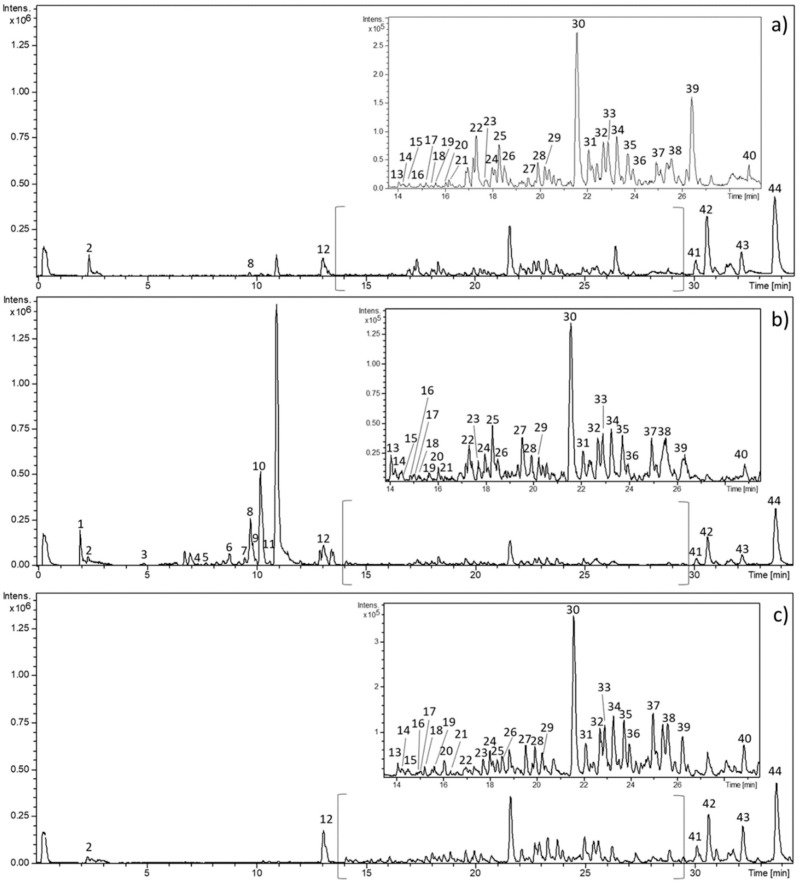
Base peak chromatogram of representative maceration (M 13) (**a**), pressurized liquid (PLE) (PLE 5) (**b**), and supercritical fluid (SFE) (SFE 1) (**c**) extracts obtained by high-performance liquid chromatography coupled with electrospray time-of-flight mass spectrometry (HPLC-ESI-QTOF-MS).

Comfrey root extracts obtained by maceration, PLE, and SFE were analyzed by HPLC-ESI-QTOF-MS (Figure 1). The identification of the compounds was based on MS spectra interpretation, considering data previously reported in the literature and open-access mass-spectral fragmentation pattern databases.

Table 1 lists the main peaks detected according to increasing retention time together with experimental and calculated *m/z*, molecular formula, error (*ppm*) value, and fragmentation pattern. A total of 44 compounds were tentatively identified in comfrey root belonging to various metabolite families that included anthraquinones, organic, phenolic and fatty acids, and their derivatives. Some of these compounds have been previously reported in comfrey root, however, the high-resolution of QTOF-MS enabled identification of 39 phytochemicals that have never been reported in this sample matrix.

#### 2.1.1. Organic Acids

According to mass spectra and the elution profile, only one compound was characterized as organic acid. Compound eluted as Peak 2 with *m/z* 191.0197 was tentatively assigned to citric acid that gave a fragment at *m/z* 111.0062 corresponding to the loss of H_2_O and CO_2_ molecules [M-H-CO_2_-2H_2_O]^−^. The compound is reported in *S. officinale* for the first time.

#### 2.1.2. Phenolic Acids and Derivatives

The occurrence of two hydroxycinnamic acid compounds was observed in comfrey root extracts. Compounds eluted as Peaks 1 and 5 gave [M-H]^−^ ions at *m/z* 377.0878 and 179.035, being characterized as caffeic acid derivative and caffeic acid, respectively, based on their fragmentation. The individual MS^2^ base peaks at *m/z* 341.1074 and 135.046 resulted from the loss of a CO_2_ moiety from corresponding deprotonated ion. The presence of caffeic acid in comfrey root was also previously reported by Roman et al. [9] applying HPLC-UV-Vis. Peak 3 presenting a [M-H]^−^ ion at *m/z* 299.0772, giving the indication of hydroxybenzoic acid glucoside. The molecular ion peak produced fragmented peak at *m/z* 137.0189 that matched with *p*-hydroxybenzoic acid after the loss of the glucose moiety. Compound eluting as Peak 4 displayed a [M-H]^−^ at *m/z* 137.0244 and was considered to be hydroxybenzoic acid. The parent ion peak was subjected to fragmentation that gave rise to a daughter ion at *m/z* 108.0215. The typical loss of CO_2_ was observed giving [M-H-44]^−^ as a characteristic ion at *m/z* 92.0265. Compound eluted as Peak 6 showed the pseudomolecular ion at *m/z* 537.1038, which corresponded to the structure of salvianolic acids H/I or lithospermic acid A. It was tentatively assigned to salvianolic acid H/I due to the presence of a fragment *m/z* 339, as reported in the literature [23]. This was also confirmed by other daughter ions with *m/z* 197.0437, 295.0595, and 135.0429. Compounds eluting as Peaks 7 and 9 represented salvianolic acid B isomers by a deprotonated molecular ion with *m/z* 717.1461. It was, then, authenticated by matching the fragmentation profile to a previously published report by Liu et al. [23]. The ESI-MS/MS spectra of the [M-H]^−^ ion exhibited fragments derived from the neutral loss of two molecules of danshensu (DSS) (198 u) and caffeic acid (CA) (180 u), resulting in the fragment ions at *m/z* 475.1057 (-CA-CO_2_) and 339.0527 (-DSS-CA). Peak 10 showed a molecular ion [M-H]^−^ at *m/z* 719.1618 releasing main fragment ions at *m/z* 359.075 and 161.0222 corresponding to rosmarinic acid ([M-2H]^2−^) and its deprotonated caffeoyl residue, respectively. These characteristics coincide with those reported by Barros et al. [17] for sagerinic acid, a dimer of rosmarinic acid. The dimerization occurs by a [2+2] union of the olefinic moieties (cyclobutane structure) [24].

#### 2.1.3. Fatty Acids and Derivatives

In analyzed extracts, 34 fatty acids were detected, including unsaturated, saturated, and hydroxy fatty acids. Fatty acid derivatives occur naturally as components of vegetal lipids forming part of the vegetal epidermis, but also as metabolic products of fatty acids since they are formed by oxidation [25,26]. Among these compounds, hydroxy fatty acids are biologically active exhibiting antibacterial, anti-inflammatory, and antiproliferative activities [27,28,29]. Compound eluted as Peak 11 gave a deprotonated molecule at *m/z* 187.0976 tentatively assigned to nonanedioic acid, a saturated dicarboxylic fatty acid with nine carbons. This fatty acid is found naturally in some plants playing a role in plant systemic immunity and being involved in priming defenses [30]. Its MS^2^ spectrum yielded fragment ions at *m/z* 125.0974 [M-H-H_2_O-COO]^−^ and *m/z* 97.0658 [M-H-H_2_O-COO-CO]^−^ by the loss of one of the acid groups and further dehydration. Peaks 12,13, and 16 with a [M-H]^−^ ion at *m/z* 329.2333 exhibited fragment ions at *m/z* 211.1256 [M-H-C_6_H_14_O_2_]^−^ and 199.131 [M-H-C_7_H_14_O_2_]^−^ which was characteristic for trihydroxy-octadecenoic acid [31]. Compounds eluting as Peaks 14 and 15 identified by a molecular formula C_18_H_32_O_5_, presented a MS^2^ pattern with three ions at *m/z* 209.119, 171.1031, and 185.1189. These compounds were assumed to be trihydroxy-octadecadienoic acid isomers, as reported in previous studies [32]. A loss of two water molecules from compounds eluted as Peaks 12, 13, 16, and 14, 15 suggest extra hydroxyl groups, while a mass difference of 2 amu between them indicated an extra double bond [33]. Compounds eluted as peaks 17, 19, 22, and 23 with a [M-H]^−^ ion at *m/z* 313.2384 were identified as belonging to dihydroxy-octadecenoic acid isomers by a comparison of their fragmentation patterns and bibliographic information [34,35]. Compounds eluting as Peaks 18 and 20 showed a mass of 287.2228 with a predicted molecular formula of C_16_H_32_O_4_ and a fragment ion at *m/z* 269.2123 (loss of H_2_O), tentatively identified as dihydroxyhexadecanoic acid isomers. Compounds (Peaks 21, 28, and 29) with a precursor ion at *m/z* 309.2071 were tentatively assigned to hydroperoxy-octadecatrienoic acid isomers on the basis of their fragmentation pattern which encompassed fragments at *m/z* 99.0813, 209.1183, 185.118, 171.1043, and 137.0981, and literature data [32,36]. Two isomers of hydroperoxides of octadecadienoic acid at *m/z* 311.2228 (Peaks 24 and 25) were detected. Major MS^2^ fragment was produced by the breakage of the weakest bond in the molecule, in the vicinity of the functional group, followed by a loss of water molecule. The other two isomers of hydroxy fatty acids identified in comfrey root extracts were dihydroxystearic acid isomers (Peaks 26 and 27). Their fragments yielded at *m/z* 297.2433 and 141.1286. Compounds eluting as Peaks 30, 31, 37, 38, and 39 showed a mass of 295.2279 and presented the fragment ions at *m/z* 277.2177 and 171.1026, resulting from a neutral loss of water and the cleavage of the C-C bond adjacent to the hydroxyl group, respectively. These compounds were tentatively identified as hydroxy-octadecadienoic acid isomers. Compounds eluting as Peaks 32, 33, 34, and 35 with a precursor ion at *m/z* 293.2122 were characterized as oxo-octadecadienoic acid isomers. The MS^2^ spectra showed characteristic ions at *m/z* 185.1158 and 113.0973, formed by cleavage of the double bond adjacent to the carbonyl group. Furthermore, another fragment ion at *m/z* 125.0947 resulted from the ion at *m/z* 185.1158 by loss of CH_3_COOH. Two isomers of ricinoleic acid were detected at *m/z* 297.2435 (Peaks 36 and 40), which produced MS/MS ions at *m/z* 155.1079, 279.2334, and 171.1025 [37]. The product ion spectra of *m/z* 297 of ricinoleic acid isomers, both displayed a fragment at *m/z* 279, i.e., a neutral loss of H_2_O. Compounds (Peaks 41 and 42) with precursor ions [M-H]^−^ at m/z 277.2173 were unambiguously identified as linolenic acid isomers based on our previous study [38]. Moreover, two monounsaturated and polyunsaturated fatty acids (Peaks 43 and 44) were suggested as palmitoleic acid and linoleic acid, based on the high-resolution masses (*m/z* 253.2173 and 279.233, respectively) and predicted molecular formulas [38].

#### 2.1.4. Other Phytochemical Compounds

As far as other phytochemical compounds are concerned, one anthraquinone was found in the extract. Compound eluted as Peak 8 displayed a [M-H]^−^ at *m/z* 311.0561 and was considered to be acetyl-monomethyl-trihydroxy anthraquinone. This compound has been described previously in the literature [39]. This assignment was supported by the fragment ion produced in MS/MS spectra at *m/z* 267.0667, resulting from the loss of CO_2_ moiety from corresponding deprotonated ion.

### 2.2. Comparison of Extraction Techniques

In relation to the extraction of phytochemicals from comfrey root, different extraction techniques and analytical approaches have been applied previously. Trifan et al. [12] applied HPLC-DAD-QTOF-MS/MS to characterize comfrey root extract obtained by maceration using 65% ethanol. The authors revealed the presence of rosmarinic acid that was the prominent phenolic acid, caffeic and salvianolic acids A, B, C, and I. In another report [10], aqueous extracts of comfrey root were characterized by UHPLC-DAD–HESI–MS analysis. The extracts were rich in phenolic acids, flavonoids, and allantoin. Furthermore, the profile of comfrey root phenolic compounds has been reported based on HPLC-DAD and HPLC-ED [11,21,22]. In the study by Neagu et al. [21], extraction of phytochemicals from comfrey root was carried out by maceration using 50% ethanol and 50% methanol. Vladić et al. [40] compared polyphenolic content in extracts obtained by conventional and modern extraction techniques (subcritical water extraction and ultrasound assisted extraction) and found that SWE was much more efficient for the extraction of polyphenols from *S. officinale* root. Polyphenolic profiles were also observed in methanolic and aqueous extracts of comfrey root obtained by Soxhlet extraction and decoction method [20]. Chin et al. [41] extracted tannins from lateral and main comfrey root tissue by ultrasound assisted extraction. According to the available literature, there are no reports of the use of PLE and SFE for the recovery of phytochemicals from *S. officinale* root. The coupling of the HPLC technique with a high-resolution QTOF-MS allowed the identification of a great number of phytochemicals in obtained extracts.

Table 2 shows extraction yields for applied extraction technique at different operational conditions. The extraction yield (EY) was expressed as the mass of obtained dry extract (g) per g of dry plant material, i.e., percentage (%). In maceration, the yield was highly dependent on the solvent. On the one hand, greater extraction efficiencies were obtained with solvent mixtures (methanol, ethanol, or acetone/water) as compared with pure solvents. The methanol-water mixture was the most suitable solvent for the extraction of target analytes producing the highest extraction yield (21.63%). On the other hand, the use of pure acetone significantly decreased the extraction efficiency (0.58%), probably due to the lower dielectric constant of the solvent and, consequently, poor recovery of more polar compounds. Furthermore, there were no significant differences between extraction efficiency at 30 min, 60 min, and 12 h.

As shown in Table 2, PLE was more efficient than SFE and maceration, producing significantly higher yields in a shorter time. Higher temperatures and intermediate concentrations of ethanol produced the highest EY (PLE 9, 49.92%) which was two-fold higher in comparison to the extraction yield by maceration applying the best conditions (M 4). With PLE, the desorption kinetics are greatly accelerated. The application of high temperatures enhances analyte solubility and mass transfer rates. Furthermore, the decreased solvent polarity for these experimental conditions enabled the transfer of a large number of compounds into the solvent, increasing extraction yield. The limitation of higher temperatures is due to the possible degradation of certain compounds. Hydrolysis, oxidation, methylation, isomerization, decarboxylation, enolization, and other decomposition reactions that take place in pressurized liquid extraction depend on both the temperature applied and the structure of the molecules, as well as the duration of the process [42]. On the one hand, some authors have indicated the formation of unwanted compounds as a result of thermo-oxidation, caramelization, and Maillard reactions [43]. On the other hand, at higher extraction temperatures, new bioactive compounds with significant antioxidant, antibacterial, and antihypertensive properties can be generated through thermo-oxidation, caramelization, and Maillard reactions [44,45].

Table 2 also shows that the efficiency of SFE was lower as compared with the other two techniques. SFE extraction at 40 °C, at 300 bar and using 7% ethanol as a modifier enabled the highest recovery of all applied SFE conditions. According to Hamburger et al. [46], an increase in pressure promoted an increase in the density of supercritical CO_2_, increasing its solvation power and resulting in a higher EY. By comparing all extraction techniques, the lowest yield was noted for SFE with the addition of 7% ethanol as a cosolvent at 150 bar and 40 °C.

**Figure 2:**
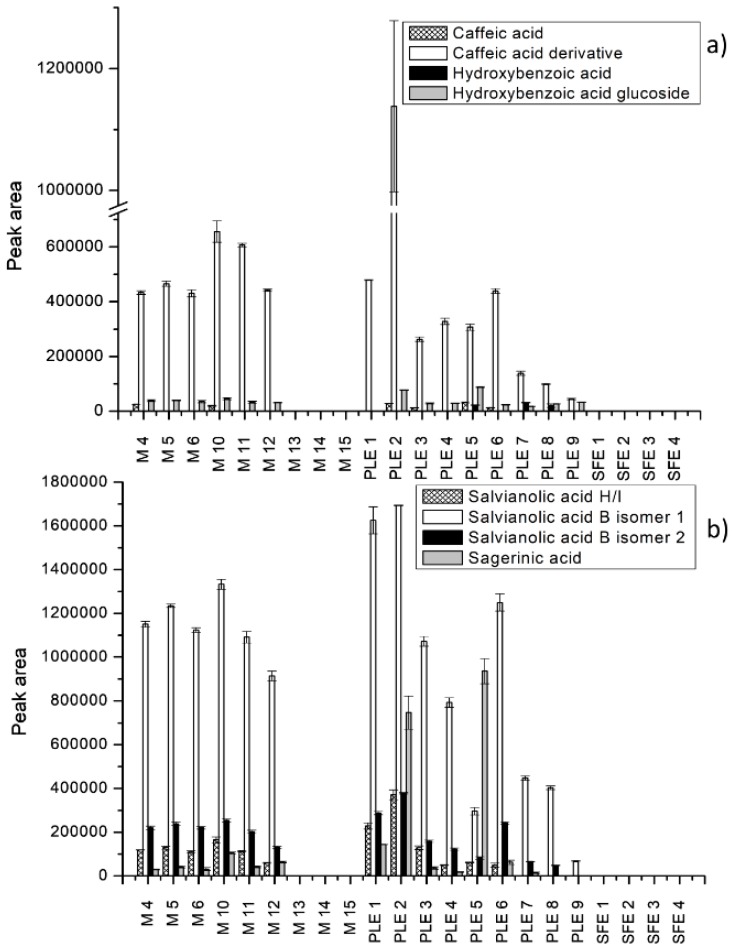
Extraction efficiency of different extraction techniques expressed as peak areas of phenolic acids. (**a**) Caffeic acid, caffeic acid derivative, hydroxybenzoic acid, and hydroxybenzoic acid glucoside; (**b**) salvianolic acid H/I, salvianolic acid B isomers, and sagerinic acid.

**Figure 3:**
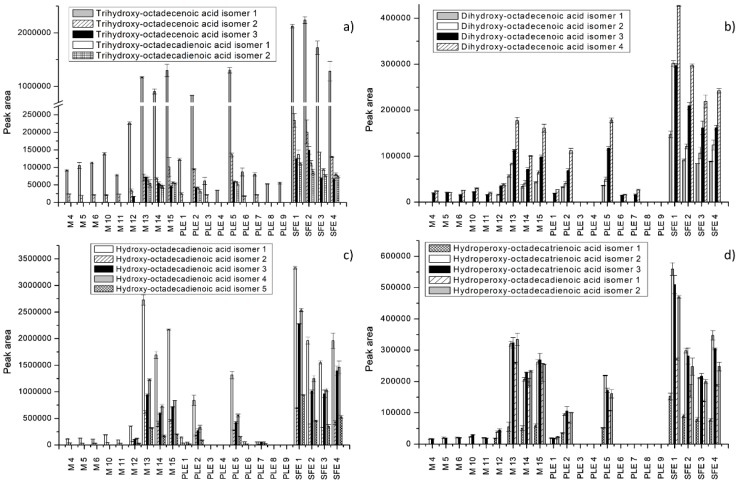
Extraction efficiency of different extraction techniques expressed as peak areas of some fatty acids and derivatives. (**a**,**b**) Trihydroxy-octadecenoic acid isomers, trihydroxy-octadecadienoic acid isomers, and dixydroxy-octadecenoic acid isomers; (**c**,**d**) hydroxy-octadecadienoic acid isomers, hydroperoxy-octadecatrienoic acid isomers, and hydroxy-octadecadienoic acid isomers.

**Figure 4:**
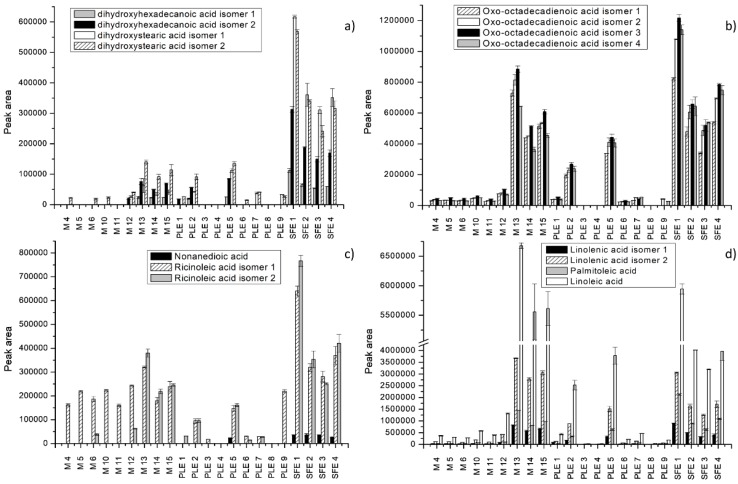
Extraction efficiency of different extraction techniques expressed as peak areas of some fatty acids and derivatives. (**a**,**b**) Dixydroxyhexadecenoic acid isomers, dixydroxystearic acid isomers and oxo-octadecadienoic acid isomers; (**c**,**d**) nonanedioic acid, ricinoleic acid isomers, linolenic acid isomers, palmitoleic, and linoleic acid.

Moreover, the comparison of extraction efficiency was carried out by comparing peak areas of the identified compounds. Extraction efficiency was depicted for each extraction technique at different operational conditions for all identified individual compounds (Figure 2, Figure 3 and Figure 4). The peak areas of the identified compounds in *S. officinale* root extracts were expressed as mean ± standard deviation of the three analyses replicates (Table S1). The significant differences between extraction techniques in terms of contents of individual compounds were analyzed statistically (Table S2). Results of the performed analysis suggested that there were statistically significant differences in the recovery of compounds in extracts obtained by maceration, PLE, and SFE.

The characterization of extracts was carried out only for selected extracts obtained by maceration and for the most efficient extracts obtained by PLE and SFE (M 4 to 6 and 10 to 15, PLE 1 to 9, and SFE 1 to 4). As observed in Figure 2, Figure 3 and Figure 4, the greatest number of identified compounds was seen in extracts obtained by PLE.

Results showed that the recovery of citric acid was similar in all tested extracts, suggesting a minor influence of the extraction technique. The macerate using 75% ethanol during 30 min was the richest in citric acid.

As shown in Figure 2, phenolic acids and derivatives were not extracted by maceration using acetone and SFE, suggesting that more polar solvents such as ethanol and methanol are necessary for the extraction of phenolic compounds. With respect to individual phenolic acids, the recovery of salvianolic acid B isomer 1 was found to be the highest in the extracts obtained by PLE and macerates using 75% EtOH and MeOH (Figure 2b). The highest content was observed for the extract obtained by PLE using 85% EtOH at 63 °C. In this extract, the content of salvianolic acid B isomer 2 was more than four-fold higher than the contents of salvianolic acid B isomer 2 in all extracts. Similar results concerning salvianolic acid B content in *S. officinale* macerates obtained with 65% EtOH have been previously reported [12]. Slightly lower peak areas were seen for caffeic acid derivative at the same experimental conditions (Figure 2a). For the rest of phenolic compounds, PLE using 85% ethanol at 63 °C was the most appropriate. Tahirovic et al. [11] also identified caffeic acid in comfrey root macerates obtained by water in lower concentration as compared with other phenolic acids. The presence of caffeic acid in macerates (70% EtOH, seven days) was also confirmed by Roman et al. [9]. By contrast, the recovery of hydroxybenzoic acid was more efficient by PLE using 85% EtOH at 176 °C, while sagerinic acid was extracted better by PLE using 100% EtOH at 120 °C. Moreover, by comparing peak areas of phenolic compounds in the extracts obtained by PLE at different temperatures, we observed that an increase of the extraction temperature led to a decrease of the extraction efficiency. In contrast, concerning maceration and different alcohol-based mixtures and extraction times, there was no significant difference between the extracts in terms of the contents of phenolics.

The content of acetyl-monomethyl-trihydroxy anthraquinone significantly differed in all extracts, reaching the highest values for PLE extracts obtained with 100% EtOH at 120 °C and 85% EtOH at 63 °C. Optimal macerate for this compound (M 12) showed four-fold lower recovery, whereas in general all SFE extracts were very poor in this anthraquinone.

Fatty acids were the most abundant in *S. officinale* root extracts. As shown in Figure 3 and Figure 4, the extracts obtained by SFE and maceration using acetone showed recoveries well above the values reported for other extracts. Slightly lower values were achieved by PLE using 100% ethanol at 120 °C. In the study conducted by Mani et al. [47], acetone was successfully applied for the extraction of lipids from moringa seed kernel. The results were similar to those obtained by nonpolar solvents such as hexane and petrol-ether. Some fatty acids, such as dihydroxy-octadecenoic acid isomers, hydroperoxy-octadecatrienoic acid isomers, hydroxy-octadecadienoic acid isomersdihydroxy-octadecenoic acid isomers, and dihydroxyhexadecanoic acid isomers, were not detected under specific extraction conditions. Linoleic acid was the most abundant and was detected in all extracts (Figure 4d). Other dominant fatty acids were linolenic acid, followed by hydroxy-octadecadienoic acid, trihydroxy-octadecenoic acid, and palmitoleic acid. Extraction with 100% acetone for 30 min (M 13) enabled the best recovery of trihydroxy-octadecadienoic acid isomer 1, linolenic acid isomer 1, and linoleic acid, whereas hydroxy-octadecadienoic acid isomer 1 and palmitoleic acid were found in the highest concentration in the SFE extract (7%, 150 bar). Trihydroxy-octadecenoic acid isomers 1 and 3 were best extracted by SFE using 15% EtOH as a cosolvent, at 150 bar (Figure 3a). Nonanedioic acid was present in low quantities and was extracted only by PLE (5) and SFE (Figure 4c). In fact, concerning fatty acid derivatives, extraction using water-based binary mixtures proved to have poor recovery of these compounds.

## 3. Materials and Methods

### 3.1. Chemicals and Reagents

All chemicals were of analytical reagent grade. Methanol, ethanol, and acetone used for the extraction was purchased from Fisher Scientific (Madrid, Spain). Cellulose filter and sea sand used for PLE and SFE were purchased from Fisher Scientific (Madrid, Spain). For mobile phase preparation, formic acid and acetonitrile were purchased from Fluka, Sigma-Aldrich (Steinheim, Germany), and Fisher Scientific (Madrid, Spain), respectively. Sodium carbonate was from Panreac (Barcelona, Spain). For analytical purpose, gallic acid, used as internal standard, were obtained from Sigma-Aldrich (Steinheim, Germany). Double-deionized water with conductivity lower than 18.2 MΩ was obtained with a Milli-Q system (Millipore, Bedford, MA, USA).

### 3.2. Plant Material

The commercial samples of dry *S. officinale* L. roots were purchased from a local healthy food retail store in Novi Sad, Serbia. The roots were finely ground into uniform powder using an Ultra Centrifugal Mill ZM 200 (Retsch GmbH, Haan, Germany) equipped with 12-tooth rotor and ring sieve with a trapezoid hole of 1 mm at 6000 rpm. The resulting comfrey root powder was kept at room temperature and darkness until use.

### 3.3. Sample Preparation

#### 3.3.1. Maceration

For the maceration process, a general procedure was applied which consisted of placing 5 g of comfrey root powder in a stoppered container with 20 mL of solvent. Methanol, ethanol, and acetone were used as absolute and in mixtures with water in different ratios (100:0, 75:25, and 50:50 *v*/*v*). The mixture was left to macerate for 30, 60 min, and 12 h under stirring at room temperature and, then, was centrifuged (Heraeus Sepatech Labofuge 200, Thermo Fisher Scientific Inc., Waltham, MA, USA) for 15 min at 7000 rpm. The supernatant was separated, evaporated using a rotary vacuum evaporator model R-200 coupled to a heating bath model B-490, both from Büchi Labortechnik (9230 Flawil, Switzerland) and the extracts were stored at −20 °C until the analyses. All extractions were done in triplicate.

#### 3.3.2. Pressurized Liquid Extraction

The PLE was carried out using a Dionex ASE 350 Accelerated Solvent Extractor (Dionex Corp., Sunnyvale, CA, USA). The cells were equipped with a stainless-steel frit and a cellulose filter at the bottom to avoid passage of suspended particles into the collection vial. The PLE experiments were performed in a static mode at 1500 psi for 20 min with different ethanol:water mixtures from 0% to 100%) and temperatures (from 40 to 200 °C) to cover a wide range of dielectric constant. Dried comfrey root (6 g) was mixed with 12 g of sand and loaded into a 34 mL stainless steel extraction cell. The extraction conditions described above were applied and the extracts were collected in vials. The residual solvent was evaporated using Savant SC250EXP SpeedVac Concentrator (Thermo Scientific, Sunnyvale, CA, USA) and dried extracts were stored at −20 °C protected from light until analysis.

#### 3.3.3. Supercritical Fluid Extraction

SFE was carried out with a Waters Prep Supercritical Fluid Extraction system (SFE-100) (Waters®, TharSFC, Thar Technologies, Inc., Pittsburgh, PA, USA) equipped with a high-pressure CO_2_ P-50 pump, a high pressure cosolvent P-50 pump, an automated back pressure regulator, a low-pressure heating exchanger, a high-pressure heating exchanger, a high-pressure extraction vessel, and a high-pressure collection vessel. All SFE extractions were performed at 40 °C in a dynamic mode with a total flow rate of 22 g/min of the solvent (CO_2_ plus ethanol at 7 and 15%) and pressures (150 and 300 bar). Comfrey root (5 g) were mixed with sea sand in a ratio 1:2 (m:m), placed in the extraction cell, and pressurized with CO_2_. The extract was continually transferred from the extraction vessel to a fraction collector. The back-pressure regulator allowed controlled depressurization to separate compounds of interest. The total extraction time was 120 min for each extraction. The collected extracts were concentrated in a water bath at 40 °C using a rotary evaporator. All extractions were done in triplicate.

### 3.4. HPLC-ESI-QTOF-MS Analysis

The extracts were reconstituted in different amounts of the extraction solvent depending on the quantity of dry extract, up to a concentration of 1000 mg/L. The final extracts were filtered through 0.2 μm nylon syringe filters (Millipore, Bedford, MA, USA). To define the phytochemical profile of comfrey root extracts, HPLC-ESI-QTOF-MS analysis was applied. Separation of the compounds was performed using an Agilent 1200 Series Rapid Resolution LC system (Agilent Technologies, Palo Alto, CA, USA) (Agilent Technologies, Palo Alto, CA, USA) of the Series Rapid Resolution coupled to an electro-spray quadrupole time-of-flight mass spectrometer, previously described by Nastić et al. [38]. The mobile phase was 0.1% formic acid in water as eluent A and acetonitrile as eluent B. The solvent gradient changed according to the following conditions: 0 min, 5% B; 15 min, 65% B; 36 min, 95% B; 40 min, 5% B, and, then, a conditioning cycle of 5 min with the initial conditions. External mass-spectrometer calibration was performed using a 74900-00-05 Cole Palmer syringe pump (Vernon Hills, Illinois, USA) directly connected to the interface, equipped with a Hamilton syringe (Reno, Nevada, USA) containing sodium formate clusters (5 mM sodium hydroxide in water:2-propanol 1:1 (*v*/*v*), with 0.2% of formic acid) in quadratic high-precision calibration (HPC) regression mode. The calibration solution was injected at the beginning of each run and all spectra were calibrated prior to identification of compounds.

### 3.5. Statistical Analysis

Origin (Version Origin Pro 8.5, Northampton, MA, USA) was employed to perform one-way analysis of variance (ANOVA) at a 95% confidence level (*p* ≤ 0.05) in order to analyze statistically significant differences between extraction techniques in terms of contents of phytochemicals.

## 4. Conclusions

In the present work the efficiency of three different extraction techniques (maceration, PLE, and SFE) for the recovery of phytochemicals from comfrey root was compared. A detailed characterization of phytochemical profiles of the comfrey root extracts was carried out by HPLC-ESI-QTOF-MS/MS. In analyzed extracts, 44 comfrey metabolites were separated and identified, including 39 newly discovered compounds. More than 50% of these compounds corresponded to fatty acids, followed by organic and phenolic acids and their derivatives. Different extraction conditions were tested for the most efficient recovery of phytochemicals from *S. officinale* root. PLE allowed a high recovery of a wide range of phytochemicals of different polarities. In general, PLE is recommended for the extraction of more polar compounds, while SFE shows to be more efficient in the recovery of less polar compounds. Furthermore, the use of acetone-based solid–liquid mixtures produced higher recoveries of nonpolar compounds, while the use of alcohol-based solvents (aqueous ethanol or methanol) produced a higher recovery of all phenolic compounds. The main compositional differences between extracts obtained by different extraction techniques were assigned to the solvent type. This work provided useful information with respect to the best extraction conditions for each *S. officinale* root compound or family of compounds.

## Figures and Tables

**Table 1:** Proposed compounds tentatively identified in *S. officinale* root extracts obtained by different extraction techniques using HPLC-ESI-QTOF-MS. Numbers designing compounds correspond to peaks as depicted in Figure 1.

Peak	Retention Time (min)	*m/z* Experimental	*m/z* Calculated	(M-H)^-^	Error (*ppm*)	Fragment m/z (Relative Abundance)	Proposed Compound	Extracts **
1	1.98	377.0876	377.0878	C_18_H_17_O_9_	0.4	341.1074 (3.49%)	caffeic acid derivative *	M 4–6, 10–12, PLE 1–9
2	2.33	191.0188	191.0197	C_6_H_7_O_7_	5.0	111.0062 (15.15%)	citric acid *	M 4–6, 10–15 PLE 1–9, SFE 1–4
3	4.83	299.0767	299.0772	C_13_H_15_O_8_	1.7	93.0303 (100%); 137.0189 (38.79%)	hydroxybenzoic acid glucoside *	M 4–6, 10–12 PLE 1–9
4	7.48	137.0226	137.0244	C_7_H_5_O_3_	13.6	108.0215 (67.17%); 92.0265 (38.19%)	hydroxybenzoic acid	PLE 5, 7,8
5	7.96	179.0332	179.035	C_9_H_7_O_4_	9.9	135.046 (100%); 107.0505 (5.69%)	caffeic acid	M 4,10, PLE 2,3,5,6
6	8.76	537.1036	537.1038	C_27_H_21_O_12_	0.4	197.0437 (100%); 339.0496 (86.75%); 295.0595 (70.42%); 135.0429 (69.26%)	salvianolic acid H/I	M 4–6, 10–12, PLE 1–6
7	9.48	717.1483	717.1461	C_36_H_29_O_16_	−3.0	475.1057 (100%); 339.0527 (83.44%)	salvianolic acid B isomer 1	M 4–6, 10–12, PLE 1–9
8	9.70	311.0565	311.0561	C_17_H_11_O_6_	−1.4	267.0667 (100%)	acetyl-monomethyl-trihydroxy anthraquinone *	M 4–6, 10–15, PLE 1–9, SFE 2,3
9	9.87	717.1458	717.1461	C_36_H_29_O_16_	0.5	475.1128 (100%); 339.0568 (46.94%)	salvianolic acid B isomer 2	M 4–6, 10–12, PLE 1–8
10	10.17	719.163	719.1618	C_36_H_31_O_16_	−1.7	161.0222 (100%); 359.075 (37.78%); 197.0432 (31.09%)	sagerinic acid *	M 4–6, 10–12, PLE 1–7
11	10.30	187.0963	187.0976	C_9_H_15_O_4_	6.8	125.0974 (100%); 97.0658 (54.77%)	nonanedioic acid *	PLE 5, SFE 1–4
12	13.03	329.2339	329.2333	C_18_H_33_O_5_	−1.7	211.1256 (100%); 229.1358 (40.5%)	trihydroxy-octadecenoic acid isomer 1 *	M 4–6, 10–15, PLE 1–9. SFE 1–4
13	14.07	329.2344	329.2333	C_18_H_33_O_5_	−3.2	199.131 (100%); 211.1311 (67.96%); 129.0891 (43.67%)	trihydroxy-octadecenoic acid isomer 2 *	M 4–6, 10–15, PLE 1–3, 5–7, SFE 1–4
14	14.30	327.2166	327.2177	C_18_H_31_O_5_	3.2	209.119 (100%); 129.0922 (48.53%); 185.1188 (46.63%)	trihydroxy-octadecadienoic acid isomer 1 *	M 13–15, PLE 2,5, SFE 1–4
15	14.50	327.2186	327.2177	C_18_H_31_O_5_	−2.7	209.119 (100%); 171.1031 (41.88%); 185.1189 (22.87%)	trihydroxy-octadecadienoic acid isomer 2 *	M 13–15, PLE 2,5, SFE 1–4
16	15.07	329.2344	329.2333	C_18_H_33_O_5_	−3.1	201.1134 (100%); 171.1029 (90.74%); 199.134 (84.53%)	trihydroxy-octadecenoic acid isomer 3 *	M 12–15, PLE 2,5, SFE 1–4
17	15.24	313.2396	313.2384	C_18_H_33_O_4_	−3.8	127.1128 (12.62%); 99.0815 (11.69%)	dihydroxy-octadecenoic acid isomer 1 *	M 13–15, PLE 1,5, SFE 1–4
18	15.54	287.222	287.2228	C_16_H_31_O_4_	2.8	269.2123 (35.08%); 199.1342 (16.94%)	dihydroxyhexadecanoic acid isomer 1 *	M 13–15, PLE 2,5, SFE 1–4
19	15.64	313.2383	313.2384	C_18_H_33_O_4_	0.3	99.0813 (18.71%); 127.1132 (15.64%)	dihydroxy-octadecenoic acid isomer 2 *	M 12–15, PLE 2,5, SFE 1–4
20	16.06	287.2225	287.2228	C_16_H_31_O_4_	1.0	269.2129 (39.5%); 85.0657 (13.92%)	dihydroxyhexadecanoic acid isomer 2 *	M 12–15, PLE 1,2,5, SFE 1–4
21	16.34	309.208	309.2071	C_18_H_29_O_4_	−2.7	99.0813 (100%); 209.1183 (67.85%); 185.118 (45.57%)	hydroperoxy-octadecatrienoic acid isomer 1 *	M 12–15, PLE 2,5, SFE 1–4
22	17.68	313.2357	313.2384	C_18_H_33_O_4_	8.8	183.139 (100%); 129.0921 (55.77%)	dihydroxy-octadecenoic acid isomer 3 *	M 4–6, 10–15, PLE 1,2,5–7, SFE 1–4
23	17.94	313.239	313.2384	C_18_H_33_O_4_	−1.8	201.1136 (100%); 127.113 (21.88%)	dihydroxy-octadecenoic acid isomer 4 *	M 4–6, 10–15, PLE 1,2,5–7, SFE 1–4
24	18.13	311.2237	311.2228	C_18_H_31_O_4_	−3.0	293.2105 (100%); 211.1317 (53.74%); 197.1162 (39.72%)	hydroperoxy-octadecadienoic acid isomer 1 *	M 13–15, PLE 1,2,5, SFE 1–4
25	18.51	311.2219	311.2228	C_18_H_31_O_4_	3.0	171.0966 (100%); 211.1269 (34.77%); 139.1079 (24.92%)	hydroperoxy-octadecadienoic acid isomer 2 *	M 13–15, PLE 1,2,5, SFE 1–4
26	18.83	315.2544	315.2541	C_18_H_35_O_4_	−1.0	297.2433 (23.81%); 141.1286 (15.51%)	dihydroxystearic acid isomer 1 *	M 12–15, PLE 2,5,7,9, SFE 1–4
27	19.52	315.2548	315.2541	C_18_H_35_O_4_	−2.4	297.244 (31.33%); 141.1284 (13.19%%)	dihydroxystearic acid isomer 2 *	M 4,6,10, 12–15, PLE 1,2,5–7,9, SFE 1–4
28	19.88	309.2064	309.2071	C_18_H_29_O_4_	2.3	171.1043 (100%); 137.0981 (99.09%)	hydroperoxy-octadecatrienoic acid isomer 2 *	M 4–6, 10–15, PLE 1,2,5, SFE 1–4
29	20.20	309.2067	309.2071	C_18_H_29_O_4_	1.5	139.1131 (100%); 291.1966 (34.57%); 125.0971 (27.59%)	hydroperoxy-octadecatrienoic acid isomer 3 *	M 4–6, 10–15, PLE 1,2,5, SFE 1–4
30	21.51	295.2277	295.2279	C_18_H_31_O_3_	0.4	277.2177 (100%); 171.1026 (49.67%)	hydroxy-octadecadienoic acid isomer 1 *	M 4–6, 10–15, PLE 1,2,5–7, SFE 1–4
31	22.08	295.2296	295.2279	C_18_H_31_O_3_	−5.9	277.218 (100%); 171.1031 (82.29%)	hydroxy-octadecadienoic acid isomer 2 *	M 4–6, 10–15, PLE 12,5–7, SFE 1–4
32	22.66	293.2135	293.2122	C_18_H_29_O_3_	−4.4	113.0973 (100%); 57.0345 (35.05%)	oxo-octadecadienoic acid isomer 1 *	M 4–6, 10–15, PLE 1,2,5–7, SFE 1–4
33	22.88	293.2141	293.2122	C_18_H_29_O_3_	−6.5	113.0978 (100%); 57.0346 (33.26%)	oxo-octadecadienoic acid isomer 2 *	M 4–6, 10–15, PLE 1,2,5–7,9, SFE 1–4
34	23.23	293.2136	293.2122	C_18_H_29_O_3_	−4.6	185.1158 (47.63%); 125.0947 (38.98%)	oxo-octadecadienoic acid isomer 3 *	M 4–6, 10–15, PLE 1,2,5–7,9, SFE 1–4
35	23.70	293.2137	293.2122	C_18_H_29_O_3_	−5.0	185.1183 (51.23%); 125.0973 (30.13%)	oxo-octadecadienoic acid isomer 4 *	M 4–6, 10–15, PLE 1,2,5–7,9, SFE 1–4
36	23.91	297.2447	297.2435	C_18_H_33_O_3_	−3.9	155.1079 (95.6%); 279.2334 (82.35%)	ricinoleic acid isomer 1 *	M 4–6, 10–15, PLE 2,3,5–7,9 SFE 1–4
37	25.07	295.2287	295.2279	C_18_H_31_O_3_	−3.0	139.1135 (11.07%); 277.2188 (8.11%)	hydroxy-octadecadienoic acid isomer 3 *	M 12–15, PLE 2,5,7, SFE 1–4
38	25.54	295.2308	295.2279	C_18_H_31_O_3_	−9.9	59.0144 (21.6); 125.0974 (16.49%)	hydroxy-octadecadienoic acid isomer 4 *	M 12–15, PLE 1,2,5,7, SFE 1–4
39	26.17	295.2293	295.2279	C_18_H_31_O_3_	−5.0	111.0813 (31.26%); 165.1289 (15.95%)	hydroxy-octadecadienoic acid isomer 5 *	M 12–15, PLE 1,2,5,7, SFE 1–4
40	28.78	297.2448	297.2435	C_18_H_33_O_3_	−4.3	171.1025 (36.22%); 280.2375 (17.18%)	ricinoleic acid isomer 2 *	M 6, 12–15, PLE 1,2,5–7, SFE 1–4
41	30.02	277.2171	277.2173	C_18_H_29_O_2_	0.7	59.0141 (77.82%)	linolenic acid isomer 1 *	M 4–6, 10–15, PLE 1,2,5–7,9, SFE 1–4
42	30.55	277.2191	277.2173	C_18_H_2_9O_2_	−6.6	59.0139 (100%); 83.0503 (14.38%)	linolenic acid isomer 2 *	M 4–6, 10–15, PLE 1,2,5–7,9, SFE 1–4
43	32.11	253.2169	253.2173	C_16_H_29_O_2_	1.7	191.108 (2.02%)	palmitoleic acid *	M 4–6, 10–15, PLE 1–7,9, SFE 1–3
44	33.63	279.2345	279.233	C_18_H_31_O_2_	−5.5	59.0131 (3.51%)	linoleic acid *	M 4–6, 10–15, PLE 1–9, SFE 1–4

* Compound identified in comfrey root for the first time; ** the extraction conditions of obtained extracts are defined in Table 2.

**Table 2:** Extraction yield obtained for *S. officinale* root using different extraction techniques performed in different extraction conditions.

Code	Extraction technique	Extraction solvent	Extraction conditions	Dielectric constant	EY (%)
M 1	Maceration	100% MeOH	t = 30 min		2.02
M 2	Maceration	100% MeOH	t = 60 min		2.59
M 3	Maceration	100% MeOH	t = 12 h		4.40
M 4	Maceration	75% MeOH	t = 30 min		21.63
M 5	Maceration	75% MeOH	t = 60 min		18.61
M 6	Maceration	75% MeOH	t = 12 h		21.37
M 7	Maceration	100% EtOH	t = 30 min		1.60
M 8	Maceration	100% EtOH	t = 60 min		1.50
M 9	Maceration	100% EtOH	t = 12 h		2.12
M 10	Maceration	75% EtOH	t = 30 min		12.33
M 11	Maceration	75% EtOH	t = 60 min		13.34
M 12	Maceration	75% EtOH	t = 12 h		14.30
M 13	Maceration	100% Acetone	t = 30 min		0.58
M 14	Maceration	100% Acetone	t = 60 min		0.58
M 15	Maceration	100% Acetone	t = 12 h		1.19
PLE 1	PLE	50% EtOH	T = 40 °C, t = 20 min	48.02	4.07
PLE 2	PLE	85% EtOH	T = 63 °C, t = 20 min	31.02	3.73
PLE 3	PLE	15% EtOH	T = 63 °C, t = 20 min	59.09	4.12
PLE 4	PLE	100% H_2_O	T = 120 °C, t = 20 min	19	9.04
PLE 5	PLE	100% EtOH	T = 120 °C, t = 20 min	50.41	5.50
PLE 6	PLE	50% EtOH	T = 120 °C, t = 20 min	34.71	8.84
PLE 7	PLE	85% EtOH	T = 176 °C, t = 20 min	21.55	36.33
PLE 8	PLE	15% EtOH	T = 176 °C, t = 20 min	33.43	41.90
PLE 9	PLE	50% EtOH	T = 200 °C, t = 20 min	26.00	49.92
SFE 1	SFE	CO_2_ + 7% ethanol	T = 40 °C, p = 150 bar, t = 2 h		0.51
SFE 2	SFE	CO_2_ + 15% ethanol	T = 40 °C, p = 150 bar, t = 2 h		1.24
SFE 3	SFE	CO_2_ + 7% ethanol	T = 40 °C, p = 300 bar, t = 2 h		1.57
SFE 4	SFE	CO_2_ + 15% ethanol	T = 40 °C, p = 300 bar, t = 2h		0.76

## Supplementary Materials

The following are available online. Table S1: Peak areas of the identified compounds in *S. officinale* root extracts expressed as mean ± standard deviation of the three analyses replicates, Table S2: Statistical data (ANOVA) of best extraction condition for compounds which appear in two and three extraction techniques.
