# Supplementary material for: Comparative Assessment of Phytochemical Profiles of Comfrey (Symphytum officinale L.) Root Extracts Obtained by Different Extraction Techniques
Source: Molecules. 2020 Feb 14;25(4):837. doi: 10.3390/molecules25040837 (PMC7070662; doi:10.3390/molecules25040837)
Supplement: Supplementary file 1 [file molecules-25-00837-s001.zip › Table S1.docx]

Table S1. Peak areas of the identified compounds in *S. officinale* root extracts expressed as mean ± standard deviation of the three analyses replicates.

| Proposed compound | Peak area x E+4 | | | | | | | | | | | | | | | | | | | | | |
| --- | --- | --- | --- | --- | --- | --- | --- | --- | --- | --- | --- | --- | --- | --- | --- | --- | --- | --- | --- | --- | --- | --- |
|  | M 4 | M 5 | M 6 | M 10 | M 11 | M 12 | M 13 | M 14 | M 15 | PLE 1 | PLE 2 | PLE 3 | PLE 4 | PLE 5 | PLE 6 | PLE 7 | PLE 8 | PLE 9 | SFE 1 | SFE 2 | SFE 3 | SFE 4 |
| Organic acids | | | | | | | | | | | | | | | | | | | | | | |
| citric acid | 125±3 | 112±0.8 | 111±6 | 127±0.06 | 137±16 | 134±3 | 62±2 | 68±1 | 77±2 | 93±0.3 | 125±11 | 93±0.5 | 69±0.3 | 95±8 | 104±4 | 116±10 | 75±0.8 | 97±1 | 64±0.5 | 68±1 | 88±2 | 71±2 |
| Phenolic acids and derivatives | | | | | | | | | | | | | | | | | | | | | | |
| caffeic acid derivative | 43±0.6 | 47±1 | 43±1 | 66±4 | 61±0.7 | 44±0.4 | 0 | 0 | 0 | 48±0.1 | 114±14 | 26±0.8 | 33±1 | 31±1 | 44±0.9 | 13±0.8 | 10±0.2 | 4±0.2 | 0 | 0 | 0 | 0 |
| ​hydroxybenzoic acid glucoside | 4±0.4 | 4±0.1 | 4±0.5 | 5±0.3 | 3±0.3 | 3±0.2 | 0 | 0 | 0 | 0 | 8±0.1 | 3±0.2 | 3±0.1 | 8±0.1 | 2±0.1 | 2±0.07 | 3±0.01 | 1±0.1 | 0 | 0 | 0 | 0 |
| hydroxybenzoic acid | 0 | 0 | 0 | 0 | 0 | 0 | 0 | 0 | 0 | 0 | 0 | 0 | 0 | 2±0.1 | 0 | 3±0.1 | 2±0.6 | 0 | 0 | 0 | 0 | 0 |
| caffeic acid | 3±0.003 | 0 | 0 | 2±0.2 | 0 | 0 | 0 | 0 | 0 | 0 | 3±0.04 | 1±0.08 | 0 | 3±0.2 | 1±0.05 | 0 | 0 | 0 | 0 | 0 | 0 | 0 |
| salvianolic acid H/I | 12±0.07 | 13±0.7 | 11±0.5 | 17±1 | 11±0.3 | 6±0.2 | 0 | 0 | 0 | 23±1 | 37±2 | 13±0.8 | 5±0.2 | 6±0.2 | 5±0.9 | 0 | 0 | 0 | 0 | 0 | 0 | 0 |
| salvianolic acid B isomer 1 | 115±1 | 123±0.8 | 112±1 | 133±2 | 109±3 | 91±2 | 0 | 0 | 0 | 163±6 | 169±0.2 | 107±2 | 79±2 | 30±2 | 125±4 | 44±0.9 | 40±0.8 | 7±0.4 | 0 | 0 | 0 | 0 |
| salvianolic acid B isomer 2 | 22±0.8 | 24±0.8 | 22±0.6 | 25±0.7 | 20±0.7 | 13±0.6 | 0 | 0 | 0 | 29±7 | 38±0.3 | 16±0.5 | 12±0.6 | 8±0.6 | 24±0.6 | 6±0.06 | 5±0.1 | 0 | 0 | 0 | 0 | 0 |
| sagerinic acid | 3±0.2 | 4±0.5 | 3±0.7 | 10±0.5 | 4±0.4 | 6±0.4 | 0 | 0 | 0 | 14±0.003 | 75±8 | 4±0.6 | 2±0.1 | 94±6 | 6±0.1 | 1±0.3 | 0 | 0 | 0 | 0 | 0 | 0 |
| Fatty acids derivatives | | | | | | | | | | | | | | | | | | | | | | |
| nonanedioic acid | 0 | 0 | 0 | 0 | 0 | 0 | 0 | 0 | 0 | 0 | 0 | 0 | 0 | 2±0.1 | 0 | 0 | 0 | 0 | 4±0.2 | 4±0.5 | 4±0.08 | 3±0.3 |
| trihydroxy-octadecenoic acid isomer 1 | 9±0.2 | 11±0.9 | 11±0.3 | 14±0.3 | 8±0.2 | 23±0.4 | 118±2 | 90±5 | 130±12 | 12±0.3 | 83±0.1 | 6±1 | 4±0.01 | 130±5 | 9±1 | 8±0.05 | 5±0.06 | 6±0.3 | 212±3 | 224±6 | 172±13 | 129±18 |
| trihydroxy-octadecenoic acid isomer 2 | 2±0.06 | 2±0.02 | 2±0.1 | 2±0.2 | 2±0.04 | 3±0.4 | 7±1 | 7±0.4 | 10±3 | 2±0.3 | 9±0.2 | 2±0.1 | 0 | 14±0.5 | 2±0.08 | 2±0.08 | 0 | 0 | 23±2 | 20±3 | 14±0.02 | 13±0.2 |
| trihydroxy-octadecadienoic acid isomer 1 | 0 | 0 | 0 | 0 | 0 | 0 | 6±1 | 5±0.5 | 6±0.2 | 0 | 4±0.3 | 0 | 0 | 6±0.05 | 0 | 0 | 0 | 0 | 14±1 | 11±0.8 | 9±0.2 | 8±0.3 |
| trihydroxy-octadecadienoic acid isomer 2 | 0 | 0 | 0 | 0 | 0 | 0 | 5±1 | 4±0.4 | 5±0.2 | 0 | 3±0.5 | 0 | 0 | 5±0.5 | 0 | 0 | 0 | 0 | 11±0.4 | 9±0.7 | 8±0.3 | 7±0.3 |
| trihydroxy-octadecenoic acid isomer 3 | 0 | 0 | 0 | 0 | 0 | 2±0.03 | 7±0.2 | 5±0.4 | 4±0.3 | 0 | 4±0.02 | 0 | 0 | 6±0.3 | 0 | 0 | 0 | 0 | 12±0.3 | 15±1 | 7±0.2 | 7±0.3 |
| dihydroxy-octadecenoic acid isomer 1 | 0 | 0 | 0 | 0 | 0 | 0 | 6±0.3 | 3±0.4 | 4±0.2 | 0 | 3±0.1 | 0 | 0 | 4±0.07 | 0 | 0 | 0 | 0 | 15±0.7 | 9±0.2 | 8±0.01 | 9±0.1 |
| dihydroxyhexadecanoic acid isomer 1 | 0 | 0 | 0 | 0 | 0 | 0 | 2±0.4 | 2±0.2 | 2±0.07 | 0 | 2±0.2 | 0 | 0 | 3±0.02 | 0 | 0 | 0 | 0 | 11±0.6 | 7±0.5 | 5±0.1 | 6±0.02 |
| dihydroxy-octadecenoic acid isomer 2 | 0 | 0 | 0 | 0 | 0 | 2±0.1 | 8±0.2 | 4±0.6 | 6±0.2 | 0 | 4±0.5 | 0 | 0 | 5±0.5 | 0 | 0 | 0 | 0 | 30±0.6 | 12±0.5 | 11±1 | 12±1 |
| dihydroxyhexadecanoic acid isomer 2 | 0 | 0 | 0 | 0 | 0 | 2±0.4 | 8±1 | 5±0.09 | 7±0.2 | 2±0.004 | 6±0.07 | 0 | 0 | 9±0.1 | 0 | 0 | 0 | 0 | 31±0.9 | 19±0.2 | 15±0.9 | 17±1 |
| hydroperoxy-octadecatrienoic acid isomer 1 | 0 | 0 | 0 | 0 | 0 | 2±0.1 | 6±1 | 5±0.8 | 6±0.6 | 0 | 4±0.2 | 0 | 0 | 5±0.09 | 0 | 0 | 0 | 0 | 15±1 | 9±0.5 | 8±0.6 | 8±0.5 |
| dihydroxy-octadecenoic acid isomer 3 | 2±0.1 | 2±0.08 | 2±0.2 | 2±0.1 | 2±0.06 | 3±0.3 | 11±0.2 | 7±0.4 | 10±0.4 | 2±0.05 | 7±0.4 | 0 | 0 | 12±0.4 | 1±0.1 | 2±0.1 | 0 | 0 | 30±0.4 | 21±0.7 | 16±1 | 16±0.5 |
| dihydroxy-octadecenoic acid isomer 4 | 2±0.06 | 2±0.007 | 3±0.04 | 3±0.2 | 2±0.2 | 4±0.3 | 18±0.7 | 10±0.1 | 16±1 | 3±0.02 | 11±0.5 | 0 | 0 | 18±0.5 | 2±0.09 | 3±0.1 | 0 | 0 | 43±0.1 | 30±0.3 | 22±1 | 24±0.5 |
| hydroperoxy-octadecadienoic acid isomer 1 | 0 | 0 | 0 | 0 | 0 | 0 | 26±0.3 | 20±1 | 23±3 | 2±0.04 | 7±0.3 | 0 | 0 | 11±0.09 | 0 | 0 | 0 | 0 | 27±0.3 | 17±2 | 14±0.2 | 19±0.4 |
| hydroperoxy-octadecadienoic acid isomer 2 | 0 | 0 | 0 | 0 | 0 | 0 | 33±2 | 23±0.3 | 25±0.2 | 2±0.2 | 10±0.05 | 0 | 0 | 16±1 | 0 | 0 | 0 | 0 | 47±0.5 | 25±3 | 20±0.6 | 25±1 |
| dihydroxystearic acid isomer 1 | 0 | 0 | 0 | 0 | 0 | 3±0.3 | 6±2 | 3±0.5 | 4±0.8 | 0 | 4±0.1 | 0 | 0 | 11±0.6 | 0 | 4±0.3 | 0 | 3±0.06 | 62±0.5 | 36±4 | 31±1 | 35±3 |
| dihydroxystearic acid isomer 2 | 2±0.2 | 0 | 2±0.3 | 2±0.3 | 0 | 4±0.1 | 14±0.6 | 9±0.9 | 11±2 | 3±0.03 | 9±0.9 | 0 | 0 | 14±0.6 | 2±0.1 | 4±0.1 | 0 | 3±0.3 | 57±0.5 | 34±0.5 | 24±2 | 32±3 |
| hydroperoxy-octadecatrienoic acid isomer 2 | 2±0.1 | 2±0.3 | 2±0.1 | 2±0.2 | 2±0.09 | 4±0.3 | 32±0.8 | 21±0.6 | 26±0.8 | 2±0.1 | 10±0.3 | 0 | 0 | 22±0.08 | 0 | 0 | 0 | 0 | 56±2 | 30±0.8 | 21±0.1 | 35±2 |
| hydroperoxy-octadecatrienoic acid isomer 3 | 2±0.09 | 2±0.1 | 2±0.07 | 3±0.2 | 2±0.02 | 4±0.5 | 32±2 | 23±0.07 | 27±2 | 2±0.08 | 11±1 | 0 | 0 | 17±0.7 | 0 | 0 | 0 | 0 | 51±3 | 28±3 | 22±0.9 | 30±0.3 |
| hydroxy-octadecadienoic acid isomer 1 | 12±0.6 | 13±0.4 | 11±0.2 | 19±0.4 | 10±0.5 | 35±0.6 | 273±10 | 169±7 | 217±1 | 15±1 | 84±10 | 0 | 0 | 131±7 | 6±0.2 | 6±0.2 | 0 | 0 | 333±3 | 196±7 | 155±3 | 196±15 |
| hydroxy-octadecadienoic acid isomer 2 | 4±0.3 | 3±0.2 | 3±0.3 | 5±0.1 | 3±0.2 | 7±0.4 | 62±3 | 40±5 | 46±2 | 4±0.2 | 18±2 | 0 | 0 | 28±1 | 2±0.1 | 2±0.1 | 0 | 0 | 69±0.8 | 39±0.06 | 33±0.4 | 42±4 |
| oxo-octadecadienoic acid isomer 1 | 3±0.4 | 3±0.1 | 3±0.2 | 4±0.2 | 3±0.02 | 8±0.2 | 73±2 | 44±0.04 | 52±1 | 4±0.1 | 19±1 | 0 | 0 | 34±0.07 | 2±0.07 | 3±0.1 | 0 | 0 | 82±0.9 | 48±3 | 34±0.5 | 54±0.9 |
| oxo-octadecadienoic acid isomer 2 | 4±0.2 | 3±0.05 | 3±0.009 | 5±0.2 | 3±0.1 | 7±0.8 | 82±3 | 45±0.1 | 54±0.5 | 4±0.2 | 23±1 | 0 | 0 | 41±0.3 | 2±0.3 | 5±0.08 | 0 | 4±0.2 | 108±0.2 | 61±4 | 49±3 | 70±0.3 |
| oxo-octadecadienoic acid isomer 3 | 4±0.3 | 5±0.2 | 4±0.5 | 6±0.4 | 4±0.2 | 11±0.1 | 89±2 | 51±0.5 | 61±1 | 5±0.2 | 27±1 | 0 | 0 | 44±2 | 3±0.2 | 4±0.1 | 0 | 0 | 122±2 | 66±3 | 52±4 | 79±0.7 |
| oxo-octadecadienoic acid isomer 4 | 3±0.1 | 3±0.1 | 3±0.1 | 5±0.06 | 3±0.2 | 7±0.6 | 64±0.1 | 36±1 | 45±1 | 4±0.5 | 24±2 | 0 | 0 | 41±3 | 2±0.1 | 5±0.05 | 0 | 3±0.08 | 114±3 | 64±6 | 54±0.4 | 75±3 |
| ricinoleic acid isomer 1 | 16±0.5 | 22±0.3 | 19±1.1 | 22±0.4 | 16±0.5 | 24±0.3 | 32±0.4 | 18±1 | 24±2 | 0 | 10±0.9 | 2±0.03 | 0 | 15±1 | 3±0.2 | 3±0.07 | 0 | 22±0.7 | 64±2 | 32±2 | 28±2 | 37±4 |
| hydroxy-​​octadecadienoic acid isomer 3 | 0 | 0 | 0 | 0 | 0 | 11±0.2 | 94±2 | 60±2 | 71±1 | 0 | 27±4 | 0 | 0 | 42±2 | 0 | 6±0.2 | 0 | 0 | 228±0.4 | 101±3 | 96±7 | 140±9 |
| hydroxy-​​octadecadienoic acid isomer 4 | 0 | 0 | 0 | 0 | 0 | 13±0.4 | 123±2 | 73±3 | 84±0.3 | 6±0.08 | 33±4 | 0 | 0 | 56±3 | 0 | 6±0.2 | 0 | 0 | 254±3 | 125±6 | 103±3 | 146±12 |
| hydroxy-​​octadecadienoic acid isomer 5 | 0 | 0 | 0 | 0 | 0 | 3±0.4 | 32±0.4 | 17±2 | 20±0.7 | 2±0.2 | 9±0.5 | 0 | 0 | 16±0.4 | 0 | 3±0.2 | 0 | 0 | 94±0.5 | 45±1 | 35±4 | 53±3 |
| ricinoleic acid isomer 2 | 0 | 0 | 4±0.4 | 0 | 0 | 6±0.2 | 38±2 | 22±1 | 25±0.5 | 3±0.01 | 10±0.8 | 0 | 0 | 16±0.7 | 1±0.09 | 3±0.2 | 0 | 0 | 77±2 | 35±3 | 25±0.4 | 42±4 |
| linolenic acid isomer 1 | 3±0.1 | 3±0.4 | 3±0.2 | 4±0.2 | 3±0.1 | 9±0.8 | 83±1 | 58±3 | 67±3 | 9±0.3 | 179±0.2 | 0 | 0 | 35±3 | 2±0.4 | 4±0.1 | 0 | 3±0.2 | 90±0.4 | 48±3 | 34±0.8 | 40±5 |
| linolenic acid isomer 2 | 12±0.4 | 12±0.1 | 8±0.2 | 19±0.2 | 11±0.06 | 42±0.7 | 368±2 | 279±6 | 306±8 | 13±0.9 | 87±0.1 | 0 | 0 | 152±11 | 6±0.8 | 14±0.9 | 0 | 6±0.1 | 305±4 | 164±8 | 125±4 | 171±15 |
| palmitoleic acid | 3±0.2 | 3±0.2 | 4±0.2 | 7±0.2 | 4±0.3 | 10±0.9 | 145±0.3 | 81±0.8 | 98±0.3 | 5±0.1 | 34±2 | 2±0.04 | 2±0.01 | 63±4 | 3±0.07 | 6±0.2 | 0 | 3±0.1 | 212±3 | 89±3 | 63±4 | 109±3 |
| linoleic acid | 37±1.6 | 31±0.07 | 28±0.5 | 58±1.5 | 39±1.6 | 132±2.5 | 668±5 | 556±47 | 561±28 | 44±3 | 253±20 | 3±0.02 | 3±0.04 | 379±36 | 21±0.9 | 47±0.8 | 5±0.007 | 18±0.4 | 595±9 | 402±0.7 | 320±2 | 397±39 |
| Other phytochemicals |  |  |  |  |  |  |  |  |  |  |  |  |  |  |  |  |  |  |  |  |  |  |
| acetyl-monomethyl-trihydroxy anthraquinone | 18±1 | 19±0.6 | 17±0.6 | 27±3 | 20±0.1 | 40±0.003 | 13±0.5 | 10±0.6 | 26±0.5 | 29±2 | 114±12 | 17±0.09 | 25±1 | 199±3 | 35±21 | 32±3 | 16±2 | 34±0.4 | 0 | 4±0.2 | 4±0.3 | 0 |
