# Supplementary material for: Comparative Assessment of Phytochemical Profiles of Comfrey (Symphytum officinale L.) Root Extracts Obtained by Different Extraction Techniques
Source: Molecules. 2020 Feb 14;25(4):837. doi: 10.3390/molecules25040837 (PMC7070662; doi:10.3390/molecules25040837)
Supplement: Supplementary file 1 [file molecules-25-00837-s001.zip › Table S2.docx]

Table S2. Statistical data (ANOVA) of best extraction condition for compounds which appear in two and three extraction techniques.

| Organic acids | | | | | Phenolic acid and derivatives | | | | | | | | | | | | | | | | | |
| --- | --- | --- | --- | --- | --- | --- | --- | --- | --- | --- | --- | --- | --- | --- | --- | --- | --- | --- | --- | --- | --- | --- |
| Citric acid | | | | | **Caffeic acid derivative** | | | **Hydroxybenzoic acid glucoside** | | | **Caffeic acid** | | | **Salvianolic acid H/I** | | | **Salvianolic acid B isomer 1** | | | **Salvianolic acid B isomer 2** | | |
| M 11 | PLE 2 | 0 | | | M 10 | PLE 2 | 1 | M 10 | PLE 2 | 1 | M 10 | PLE 2 | 1 | M 10 | PLE 2 | 1 | M 10 | PLE 2 | 1 | M 10 | PLE 2 | 1 |
| PLE 2 | SFE 3 | 1 | | |  |  | |  |  |  |  |  |  |  |  |  |  |  |  |  |  |  |
| SFE 3 | M 11 | | 1 | | |  | |  |  |  |  |  |  |  |  |  |  |  |  |  |  |  |
|  | | | | **Fatty acids derivatives** | | | | | | | | | | | | | | | | | | |
| Sagerinic acid | | | | | **Nonanedioic acid** | | | **Trihydroxy-octadecenoic acid isomer 1** | | | **Trihydroxy-octadecenoic acid isomer 2** | | | **Trihydroxy-octadecadienoic acid isomer 1** | | | **Trihydroxy-octadecadienoic acid isomer 2** | | | **Trihydroxy-octadecenoic acid isomer 3** | | |
| M 10 | PLE 5 | 1 | | | M 5 | SFE 2 | 1 | M 15 | PLE 5 | 0 | M 15 | PLE 5 | 0 | M 13 | PLE 5 | 0 | M 13 | PLE 5 | 0 | M 13 | PLE 5 | 0 |
|  |  |  | | |  |  |  | PLE 5 | SFE 2 | 1 | PLE 5 | SFE 1 | 1 | PLE 5 | SFE 1 | 1 | PLE 5 | SFE 1 | 1 | PLE 5 | SFE 2 | 1 |
|  |  |  | | |  |  |  | SFE 2 | M 15 | 1 | SFE 1 | M 15 | 1 | SFE 1 | M 13 | 1 | SFE 1 | M 13 | 1 | SFE 2 | M 13 | 1 |
|  | | | | | | | | | | | | | | | | | | | | | | |
| Dihydroxy-octadecenoic acid isomer 1 | | | | | **Dihydroxyhexadecanoic acid isomer 1** | | | **Dihydroxy-octadecenoic acid isomer 2** | | | **Dihydroxyhexadecanoic acid isomer 2** | | | **Hydroperoxy-octadecatrienoic acid isomer 1** | | | **Dihydroxy-octadecenoic acid isomer 3** | | | **Dihydroxy-octadecenoic acid isomer 4** | | |
| M 13 | PLE 5 | 1 | | | M 13 | PLE 5 | 0 | M 13 | PLE 5 | 1 | M 13 | PLE 5 | 0 | M 13 | PLE 5 | 0 | M 13 | PLE 5 | 0 | M 13 | PLE 5 | 0 |
| PLE 5 | SFE 1 | 1 | | | PLE 5 | SFE 1 | 1 | PLE 5 | SFE 1 | 1 | PLE 5 | SFE 1 | 1 | PLE 5 | SFE 1 | 1 | PLE 5 | SFE 1 | 1 | PLE 5 | SFE 1 | 1 |
| SFE 1 | M 13 | 1 | | | SFE 1 | M 13 | 1 | SFE 1 | M 13 | 1 | SFE 1 | M 13 | 1 | SFE 1 | M 13 | 1 | SFE 1 | M 13 | 1 | SFE 1 | M 13 | 1 |
|  | | | | | | | | | | | | | | | | | | | | | | |
| Hydroperoxy-octadecadienoic acid isomer 1 | | | | | **Hydroperoxy-octadecadienoic acid isomer 2** | | | **Dihydroxystearic acid isomer 1** | | | **Dihydroxystearic acid isomer 2** | | | **Hydroperoxy-octadecatrienoic acid isomer 2** | | | **Hydroperoxy-octadecatrienoic acid isomer 3** | | | **Hydroxy-octadecadienoic acid isomer 1** | | |
| M 13 | PLE 5 | 1 | | | M 13 | PLE 5 | 1 | M 13 | PLE 5 | 1 | M 13 | PLE 5 | 0 | M 13 | PLE 5 | 1 | M 13 | PLE 5 | 1 | M 13 | PLE 5 | 1 |
| PLE 5 | SFE 1 | 1 | | | PLE 5 | SFE 1 | 1 | PLE 5 | SFE 1 | 1 | PLE 5 | SFE 1 | 1 | PLE 5 | SFE 1 | 1 | PLE 5 | SFE 1 | 1 | PLE 5 | SFE 1 | 1 |
| SFE 1 | M 13 | 1 | | | SFE 1 | M 13 | 1 | SFE 1 | M 13 | 1 | SFE 1 | M 13 | 1 | SFE 1 | M 13 | 1 | SFE 1 | M 13 | 1 | SFE 1 | M 13 | 1 |
|  | | | | | | | | | | | | | | | | | | | | | | |
| Hydroxy-octadecadienoic acid isomer 2 | | | | | **Oxo-octadecadienoic acid isomer 1** | | | **Oxo-octadecadienoic acid isomer 2** | | | **Oxo-octadecadienoic acid isomer 3** | | | **Oxo-octadecadienoic acid isomer 4** | | | **Ricinoleic acid isomer 1** | | | **Hydroxy-octadecadienoic acid isomer 3** | | |
| M 13 | PLE 5 | 1 | | | M 13 | PLE 5 | 1 | M 13 | PLE 5 | 1 | M 13 | PLE 5 | 1 | M 13 | PLE 5 | 1 | M 13 | PLE 9 | 1 | M 13 | PLE 5 | 1 |
| PLE 5 | SFE 1 | 1 | | | PLE 5 | SFE 1 | 1 | PLE 5 | SFE 1 | 1 | PLE 5 | SFE 1 | 1 | PLE 5 | SFE 1 | 1 | PLE 9 | SFE 1 | 1 | PLE 5 | SFE 1 | 1 |
| SFE 1 | M 13 | 1 | | | SFE 1 | M 13 | 1 | SFE 1 | M 13 | 1 | SFE 1 | M 13 | 1 | SFE 1 | M 13 | 1 | SFE 1 | M 13 | 1 | SFE 1 | M 13 | 1 |
|  | | | | | | | | | | | | | | | | | | | | | | |
| Hydroxy-octadecadienoic acid isomer 4 | | | | | **Hydroxy-octadecadienoic acid isomer 5** | | | **Ricinoleic acid isomer 2** | | | **Linolenic acid isomer 1** | | | **Linolenic acid isomer 2** | | | **Palmitoleic acid** | | | **Linoleic acid** | | |
| M 13 | PLE 5 | 1 | | | M 13 | PLE 5 | 1 | M 13 | PLE 5 | 1 | M 13 | PLE 2 | 1 | M 13 | PLE 5 | 1 | M 13 | PLE 5 | 1 | M 13 | PLE 5 | 1 |
| PLE 5 | SFE 1 | 1 | | | PLE 5 | SFE 1 | 1 | PLE 5 | SFE 1 | 1 | PLE 2 | SFE 1 | 1 | PLE 5 | SFE 1 | 1 | PLE 5 | SFE 1 | 1 | PLE 5 | SFE 1 | 1 |
| SFE 1 | M 13 | 1 | | | SFE 1 | M 13 | 1 | SFE 1 | M 13 | 1 | SFE 1 | M 13 | 1 | SFE 1 | M 13 | 1 | SFE 1 | M 13 | 1 | SFE 1 | M 13 | 1 |
| Other phytochemicals | | | | | | | | | | | | | | | | | | | | | | |
| Acetyl-monomethyl-trihydroxy anthraquinone | | | | |  | | |  | | |  | | |  | | |  | | |  | | |
| M 13 | PLE 5 | 1 | | |  |  |  |  |  |  |  |  |  |  |  |  |  |  |  |  |  |  |
| PLE 5 | SFE 3 | 1 | | |  |  |  |  |  |  |  |  |  |  |  |  |  |  |  |  |  |  |
| SFE 3 | M 13 | 1 | | |  |  |  |  |  |  |  |  |  |  |  |  |  |  |  |  |  |  |
| 1 – significant difference; 0 – insignificant difference | | | | | | | | | | | | | | | | | | | | | | |
